# An Inordinate Fondness for Eukaryotic Diversity

**DOI:** 10.1371/journal.pbio.1001382

**Published:** 2012-08-28

**Authors:** Luke J. Harmon

**Affiliations:** Department of Biological Sciences, University of Idaho, Moscow, Idaho, United States of America

## Abstract

Why do some groups of organisms, like beetles, have so many species, and others, like the tuataras, so few? This classic question in evolutionary biology has a deep history and has been studied using both fossils and phylogenetic trees. Phylogeny-based studies have focused on tree balance, which compares the number of species across clades of the same age in the tree. These studies have suggested that rates of speciation and extinction vary tremendously across the tree of life. In this issue, Rabosky et al. report the most ambitious study to date on the differences in species diversity across clades in the tree of life. The authors bring together a tremendously large dataset of multicellular eukaryotes, including all living species of plants, animals, and fungi; they divide these organisms into 1,397 clades, accounting for more than 1.2 million species in total. Rabosky et al. find tremendous variation in diversity across the tree of life. There are old clades with few species, young clades with many species, and everything in between. They also note a peculiar aspect of their data: it is difficult or impossible to predict how many species will be found in a particular clade knowing how long a clade has been diversifying from a common ancestor. This pattern suggests complex dynamics of speciation and extinction in the history of eukaryotes. Rabosky et al.'s paper represents the latest development in our efforts to understand the Earth's biodiversity at the broadest scales.

## Abstract 2

Why do some groups have so many species, and others so few? This Primer examines a paper by Rabosky et al. that furthers our efforts to understand the Earth's biodiversity at the broadest scales.

J.B.S. Haldane—one of the most quotable of all evolutionary biologists—had a favorite saying about what patterns of species richness tell us about the nature of the Universe: “God has an inordinate fondness for beetles” (see [1] for more details). With this quip, Haldane is referring to the overwhelming number of beetle species on Earth. We still don't know exactly how many species of beetles there are on the Earth—perhaps around 400,000—but certainly, there are a lot.

One of the primary mysteries in macroevolution is the tremendous difference in numbers of species among different taxonomic groups. Modern systematists classify species into clades (groups of species that represent all of the descendents of a common ancestor, like turtles or arthropods). Different clades in the tree of life have dramatically different diversities. This might not be surprising—after all, species in one clade can be distinct from other species in size, energy use, and a thousand other ways. Also, some clades are much older than others. However, even when we control for differences in age by comparing sister clades—that is, pairs of clades that are each others' closest relative—we still see profound differences in number of species. For example, there are currently two living species of tuatara, a clade of lizard-like reptiles that currently inhabit small islands around New Zealand (Figure 1, left). These tuatara are the sister clade to the squamates, a clade of 7,000 species that includes all living snakes and lizards (Figure 1, right) [2]. Since these two groups are sister clades, they diverged from a common ancestor at exactly the same time (∼250 million years ago [3]). Tuataras used to be far more diverse in the past (though almost certainly not as diverse as squamates [4]), but their current diversity is dwarfed by the tremendous number and variety of snakes and lizards around the globe. Similar patterns occur across the whole tree of life. In fact, old, low diversity clades contain some of the most enigmatic species on Earth: ginkgo trees, coelacanths, tailed frogs, horseshoe crabs, and monotremes, among others. These species are sometimes called “living fossils,” although only some of them are actually thought to resemble their ancient ancestors [5].

**Figure 1:**
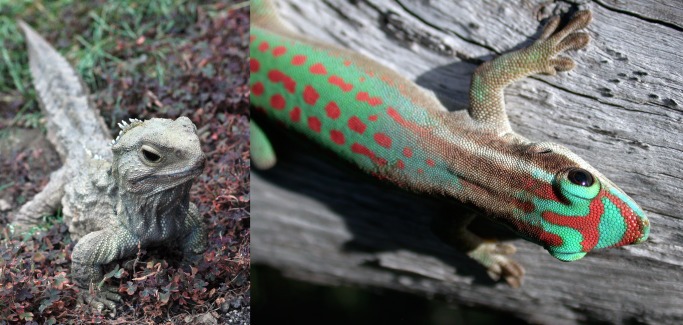
The tuatara (*Sphenodon punctatus*, left) is one of only two surviving species in the clade Sphenodontia. The sister clade to the tuatara is Squamata, which includes the ∼7,000 living species of snakes and lizards, including the ornate day gecko (*Phelsuma ornata*, right). (Left) from Wikimedia commons, taken by user KeresH, http://commons.wikimedia.org/wiki/File:Henry_at_Invercargill.jpg; (Right) by the author.

We can learn a lot about the differences in diversity across clades from phylogenetic trees. In particular, phylogenetic tree balance summarizes the pattern of differences in the number of species between sister clades across a whole phylogeny [6]. A phylogenetic tree can be completely balanced, such that each pair of sister taxa in the tree have exactly the same number of species (Figure 2A; this is only possible if the number of species in the tree is a power of 2: 2, 4, 8, 16, 32, etc.). A phylogenetic tree can also be completely imbalanced, so that every comparison of sister clades has a single species in one clade and the remainder in the other (such a tree is also called pectinate; Figure 2B). There are a few different ways to quantify tree balance, but they all work in basically the same way: compare the number of species between sister clades in the tree, and summarize those differences across a whole phylogeny [7].

**Figure 2:**
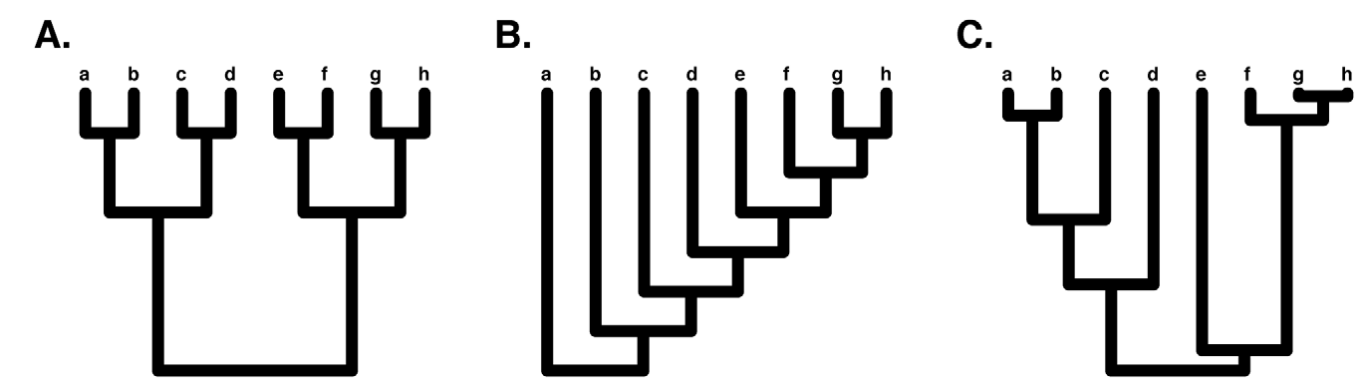
Balanced (A), imbalanced (B), and random birth-death (C) phylogenetic trees of eight species (a–h).

Imagine for a moment that you have the tree of life (a phylogenetic tree of all species on Earth). We don't have such a tree yet, but scientists are moving in that direction and trees are getting bigger and bigger (e.g., [8]). The tree of life is a huge and complicated structure, and—as one might imagine at a scale that encompasses all living things, bacteria to beetles to beagles—resists generalizations. But even as the tree of life takes shape, we already know that it is highly imbalanced. This statement applies broadly across living things, and applies equally well to plants as it does to animals, and everything else (as far as we know). Dramatic differences in diversity among clades is a characteristic feature of life on Earth.

To better understand patterns of balance in the tree of life, we can start with the birth-death model, a simple model of how phylogenetic trees might grow through time (reviewed in [9]). Under a birth-death model, phylogenetic trees “grow” through time following two processes: speciation, where one species splits into two, and extinction, when one species dies out. For simple birth-death models we assume that both of these processes happen at a constant rate through time for each species alive at that moment, and use the parameters λ (speciation rate) and μ (extinction rate). If we simulate this birth-death model using a computer, we will obtain a phylogenetic tree (Figure 2C). If the extinction rate is greater than zero, such a tree will include surviving species as well as species that have gone extinct. Since we will be comparing this tree to phylogenies of living species, we can assume that any species that went extinct before the present has been pruned. We can then measure the balance of the resulting birth-death tree (our simulated tree will also have branch lengths, but we will ignore those for now). If we repeat this process a large number of times, we obtain a statistical distribution of our tree balance measure, which represents the expectation of that distribution under the birth-death model. It turns out, perhaps surprisingly, that this distribution of balance depends only on the fact that the trees are simulated under a birth-death model; in terms of tree balance, the actual rates of speciation and extinction do not matter, as long as they are constant across clades [10].

There is another counterintuitive feature of the balance of birth-death trees: these trees are surprisingly imbalanced compared to what, perhaps, your intuition might suggest. For example, imagine that a certain phylogenetic tree contains 100 species. If you look at the deepest split in that tree and compare the diversity of the two sister clades, what do you expect to find? Are you more likely to find an even number of species in each of these two clades—say, 50/50 or 49/51—or a very uneven number, like 2/98 or 1/99? The surprising answer is that all four of these listed possibilities are equally likely. In fact, all possible combinations of diversity for each of the two clades are equally probable [11].

This property of birth-death models means that birth-death trees are quite imbalanced: it is not uncommon, for example, to find sister clades that differ in diversity by a factor of 20 just by random chance. However, the tree of life is imbalanced even compared to birth-death trees! This general observation was first discovered in an influential paper by Arne Mooers and colleagues [7]. In that paper, the authors measured the balance of phylogenetic trees that had been reconstructed using trait data, DNA sequences, or both. They then compared their balance to what we might expect under a birth-death model. They found that phylogenetic trees from a wide range of taxa are extraordinarily imbalanced. This paper showed that the general “shape” of the tree of life is highly imbalanced.

The classical interpretation of the imbalanced tree of life is that clades vary in their rates of speciation and/or extinction. There are many reasons to suspect that species in some clades might speciate more frequently, or go extinct less frequently, than their relatives. For example, perhaps a species' range affects its probability of speciating or going extinct—as suggested by a recent paper in *PLOS Biology* by Pigot et al. [12]—so that clades of species with different distributions of range sizes will experience different rates of diversification. Many studies have attempted to measure speciation and extinction rates in groups with a good fossil record, and compare these rates across different types of organisms and time periods (e.g., [13],[14]). These studies have generally found wide variation in both rates and in their difference (speciation−extinction = net diversification rate).

Recent studies go beyond measures of tree balance by using the tree's branch lengths to gain information about speciation rates. One simple way to do this is to compare the diversity of a clade to its age; one can then estimate the speciation rate as λ = *ln*(*n*)/*t*, where *n* is the number of living species in the clade and *t* is the clade's stem age (the time since divergence from the clade's sister group) [15]. There are also modifications of this equation that incorporate extinction and that can use the clade's crown age (the time since all living species in the clade shared a common ancestor; see [15]).

Perhaps surprisingly, one can even estimate extinction from a phylogenetic tree based only on living species [16]. This is because old and young lineages are hit by extinction with different probabilities: since young lineages have not been around very long, they are less likely to have gone extinct than older lineages. The phenomenon is called the “pull of the present” [16] and means that extinction leads to an overabundance of very young lineages in a tree. We can look for this pull in patterns of lineage accumulation through time, which can thus be used to estimate both speciation and extinction rates and to compare these rates across clades (e.g., [17]; but see [18], which points out that this method does not work well when its assumptions of rate constancy are strongly violated). More recently, new methods have been developed to search for variation in speciation and/or extinction rates across large phylogenetic trees, and to try to correlate these rates with the traits of lineages [19],[20].

In this issue, Rabosky et al. [21] attempt the most ambitious study to date investigating the differences in species diversity across clades in the tree of life. The authors bring together a tremendously large dataset that spans the multicellular eukaryotes, including all living species of plants, animals, and fungi. For each of 1,397 eukaryotic clades, the authors gathered estimates of age and diversity from the literature – accounting for more than 1.2 million species in total. The authors also summarize the evolutionary relationships among these clades using a “backbone” phylogenetic tree with branch lengths in millions of years. This provides a remarkably complete view of what we currently know about the species diversity of clades across a huge section of the tree of life.

The diversity data in Rabosky et al. [21] are broadly consistent with the historical background above: there are major differences in diversification rates across the tree of life. There are old clades with few species, young clades with many species, and everything in between. But Rabosky et al. also note a peculiar aspect of their data: there is typically either a very weak or no relationship between the number of species in a clade and its age. That is, in the data they analyze, it is difficult to guess how many species are in a clade on the basis of how long it has been diversifying from a common ancestor. The traditional explanation for this pattern would be differences in diversification rates across clades—although the authors use simulations to show that, at least under one scenario about how rates might vary across trees, one rarely finds such weak or absent relationships between age and diversity. The authors speculate about other possible explanations for this peculiar (lack of) pattern, from bias in the way clades are named to ecological processes that limit the number of coexisting, competing species.

Rabosky et al.'s [21] analysis is not the final chapter; the tree of life is still under construction, and the total number of species in some clades is best viewed as an educated guess. Specifically, I suspect that we have very poor estimates of the extant diversity of many eukaryotic groups, particularly small, understudied organisms. Indeed, new techniques that use genomic sequencing to identify undiscovered species from DNA sequences from environmental samples often reveal that the species we know are only a small component of natural ecosystems [22]. One might also note that the total diversity of multicellular eukaryotes counted in this study might be a vast underestimate compared to recent estimates that use statistical analyses to correct for incomplete sampling [23]. Still, the results in Rabosky et al. [21] are intriguing and will certainly inspire further study, which I expect will be focused on testing more sophisticated mathematical models, beyond the constant-rate birth-death models prevalent today, that might be able to explain patterns in the data.

I first learned of the Huxley quote that opens this article in a classic paper, “Homage to Santa Rosalia, or why are there so many kinds of animals?” [24]. This paper, written by the great ecologist G.E. Hutchinson, speculates about the mechanisms that allow so many different species to coexist in natural communities. Hutchinson describes collecting Italian water boatmen from a small pond in the shadow of Santa Rosalia's shrine, and wondering why the pond contained two species of water beetle—no more, no less. Hutchinson says “Nothing in her history being known to the contrary, perhaps for the moment we may take Santa Rosalia as the patroness of evolutionary studies…” [24]. Rabosky et al.'s [21] paper represents the latest development in our efforts to understand why the Earth has the particular number of species that it has – no more, no less. Santa Rosalia would be proud.
